# Early effects of the antineoplastic agent salinomycin on mitochondrial function

**DOI:** 10.1038/cddis.2015.263

**Published:** 2015-10-22

**Authors:** A Managò, L Leanza, L Carraretto, N Sassi, S Grancara, R Quintana-Cabrera, V Trimarco, A Toninello, L Scorrano, L Trentin, G Semenzato, E Gulbins, M Zoratti, I Szabò

**Affiliations:** 1 Department of Biology, University of Padua, Padua, Italy; 2 Department of Biomedical Sciences, University of Padua, Padua, Italy; 3 Venetian Institute for Molecular Medicine, University of Padua, Padua, Italy; 4 Department of Molecular Biology, University of Duisburg-Essen, Essen, Germany; 5 CNR Institute of Neuroscience, Padua, Italy

## Abstract

Salinomycin, isolated from *Streptomyces albus*, displays antimicrobial activity. Recently, a large-scale screening approach identified salinomycin and nigericin as selective apoptosis inducers of cancer stem cells. Growing evidence suggests that salinomycin is able to kill different types of non-stem tumor cells that usually display resistance to common therapeutic approaches, but the mechanism of action of this molecule is still poorly understood. Since salinomycin has been suggested to act as a K^+^ ionophore, we explored its impact on mitochondrial bioenergetic performance at an early time point following drug application. In contrast to the K^+^ ionophore valinomycin, salinomycin induced a rapid hyperpolarization. In addition, mitochondrial matrix acidification and a significant decrease of respiration were observed in intact mouse embryonic fibroblasts (MEFs) and in cancer stem cell-like HMLE cells within tens of minutes, while increased production of reactive oxygen species was not detected. By comparing the chemical structures and cellular effects of this drug with those of valinomycin (K^+^ ionophore) and nigericin (K^+^/H^+^ exchanger), we conclude that salinomycin mediates K^+^/H^+^ exchange across the inner mitochondrial membrane. Compatible with its direct modulation of mitochondrial function, salinomycin was able to induce cell death also in Bax/Bak-less double-knockout MEF cells. Since at the concentration range used in most studies (around 10 *μ*M) salinomycin exerts its effect at the level of mitochondria and alters bioenergetic performance, the specificity of its action on pathologic B cells isolated from patients with chronic lymphocytic leukemia (CLL) *versus* B cells from healthy subjects was investigated. Mesenchymal stromal cells (MSCs), proposed to mimic the tumor environment, attenuated the apoptotic effect of salinomycin on B-CLL cells. Apoptosis occurred to a significant extent in healthy B cells as well as in MSCs and human primary fibroblasts. The results indicate that salinomycin, when used above *μ*M concentrations, exerts direct, mitochondrial effects, thus compromising cell survival.

Salinomycin (SAL), an antibiotic that belongs to a large group of polyether ionophores, is considered to be a potential anticancer drug for cancer chemoprevention and therapy. SAL has been proved to induce programmed cell death of cancer stem cells (CSCs)^1^ as well as of various cancer cells including chronic lymphocytic leukemia (CLL) cells, human colon, breast, prostate, hepatocellular carcinoma and lung cancer cells.^2, 3, 4^ On the other hand, the use of animal models revealed that SAL has a narrow therapeutic index and SAL-induced neuropathy occurs *in vivo* even at relatively low doses, which do not give rise to systemic toxicity.^5^ Extensive research has been undertaken to clarify the general mechanism(s) by which SAL induces apoptosis and blocks tumor growth. Several independent ways of action have been proposed to account for the cancer cell-killing ability of SAL, including inhibition of proximal Wnt/*β*-catenin signaling^6^ and induction of a marked increase in the expression of the pro-apoptotic protein NAG-1.^7^ Recent results point to SAL as a strong mTORC1 signaling antagonist in breast and prostate cancer cells^8^ and as an initiator of autophagy in tumor cells.^9, 10^ SAL however is also able to suppress late stages of autophagy, leading to accumulation of dysfunctional mitochondria with increased production of reactive oxygen species (ROS).^11^

SAL is expected to impact mitochondrial function thanks to its ability to act as an ionophore. However, only few early studies addressed the direct effect of SAL on isolated mitochondria;^12^ later works generally interpreted mitochondria-related effects of SAL in intact cells presuming that SAL acts similarly to valinomycin (VAL), a potassium selective ionophore mediating potassium influx into the mitochondria according to the electrochemical driving force. For example, SAL-induced mitochondrial inner membrane (IMM) depolarization and mitochondrial ROS production have been proposed to be compatible with influx of positively charged potassium ions.^9, 13, 14^ The chemical structure of SAL is however more similar to nigericin (NIG) rather than to VAL (Supplementary Figure S1). We note the presence of a carboxyl group, which can act as the H^+^ carrier. NIG, similarly to monensin, acts as an ionophore with a preference for potassium and mediates electroneutral exchange of potassium ions with protons at the level of the IMM. Notably, NIG and monensin have been shown to induce apoptosis in human lung cancer and lymphoma cells,^15, 16^ suggesting that the common mechanism of polyether ionophore antibiotics to altering potassium homeostasis in the cytoplasm and mitochondria contributes to the induction of apoptosis in cancer cells. In accordance, we have recently demonstrated that pharmacological inhibition of a mitochondrial potassium channel (whose expression is upregulated in many cancers^17^) and the consequent mitochondrial dysfunction might be exploited to selectively kill melanoma cells even *in vivo*, as well as in *ex vivo* human leukemic cells.^18, 19^

The mitochondrial effects of SAL have generally been addressed 12–48 h following addition of the drug, even though it is expected to reach quickly the IMM, as VAL and NIG do when added to intact cells. The purpose of the present work therefore was to reveal the early effects of SAL on mitochondrial function as compared with VAL and NIG, in primary human healthy cells, in cancer stem cell-like immortalized human mammary epithelial cells expressing Twist^20^ and in cancer cells. Our results reveal the short-term effects of SAL and thus contribute to the elucidation of its mechanism of action. In light of the fact that SAL is used in screening studies on a small group of patients with invasive carcinoma of the head, neck, breast and ovary,^4^ this point is especially relevant.

## Results

**Figure 1:**
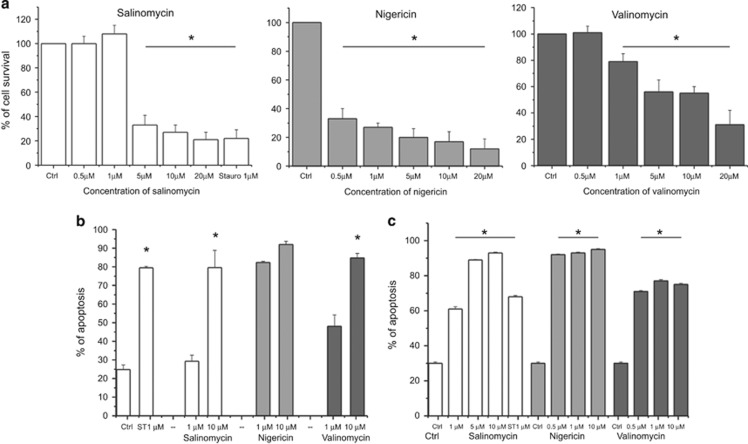
Effect of salinomycin, nigericin and valinomycin on lymphocytes. Jurkat leukemic T cells (**a** and **b**) and human primary B-CLL cells (**c**) were incubated for 24 h with different concentrations of the compounds, as indicated. Cell survival (**a**) was measured by MTT assay; values are expressed as the average percentage of cell survival compared with untreated cells ± S.E.M. (*n*=5). Cell death (**b** and **c**) was tested using FACS by the staining with FITC-Annexin V and propidium iodide. Staurosporine (Stauro or ST) was used as positive control. Indicated values refer to the percentage of dead cells ± S.E.M. (*n*=14). Statistically significant differences (*P*<0.05) are indicated by asterisks

SAL affects the cell viability of many types of solid cancer and leukemic cells. First we focused our attention on T leukemic Jurkat cells, given that alteration of mitochondrial potassium influx in these cells significantly impacts cell survival.^21, 22^ Figure 1a shows a drastic reduction of cell survival, as assessed using MTT (3-(4,5-dimethylthiazol-2-yl)-2,5-diphenyltetrazolium bromide) assay, by addition of sub-*μ*M concentration of NIG (IC_50_: 0.33 *μ*M). Similarly, Jurkat cells were sensitive to SAL and VAL in the *μ*M range (IC_50_ SAL: 3.61 *μ*M; IC_50_ VAL: 7.40 *μ*M). Figure 1b proves, using Annexin binding assay, that the decreased cell viability was due to increased apoptosis. In all 10 *μ*M of all three ionophores was sufficient to kill more than 80% of the cells, while at 1 *μ*M NIG was most effective. Next, we tested the sensitivity of *ex vivo* human cells to the above three ionophores. Human pathologic B cells isolated from patients with CLL (B-CLL) have been reported to be efficiently killed by 1 *μ*M SAL, in contrast to peripheral blood mononuclear cells from healthy subjects.^6^ In accordance, 1 and 10 *μ*M SAL induced 60% and 94% B-CLL death, respectively (Figure 1c). Similar data were obtained with NIG and with VAL (Figure 1c and Supplementary Figure S2).

**Figure 2:**
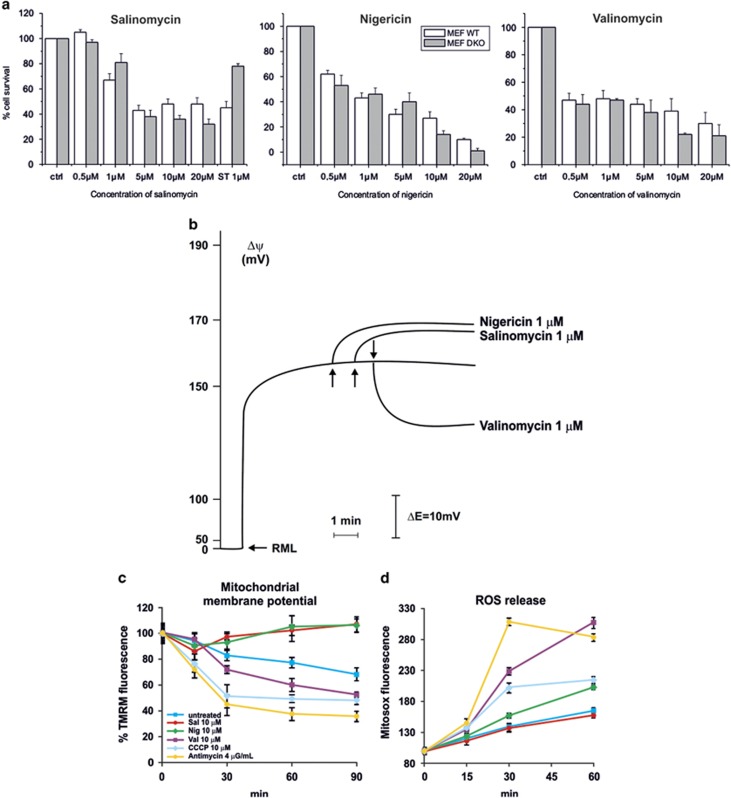
Salinomycin kills Bax/Bak-less mouse embryonic fibroblasts and alters mitochondrial membrane potential. (**a**) Cell survival in WT and Bax/Bak DKO MEFs was measured by MTT assay (*n*=4). Staurosporine was used as the classical apoptosis inducer. No significant differences were observed between WT and DKO cells. (**b**) Mitochondrial membrane potential determined on rat liver isolated mitochondria (RLM) by measuring the distribution of the TPP^+^ ions across the mitochondrial membrane with a selective TPP^+^ ion-sensitive electrode. Values are presented as variation of Δ*ψ* following addition of the indicated compounds. (**c** and **d**) Mitosox (**c**) and TMRM (**d**) fluorescence on B-CLL cells, measured by FACS analysis after treatment with salinomycin, nigericin and valinomycin at the indicated concentrations; CCCP, antimycin and staurosporine (ST) were used as positive controls. Average values ± S.D. are showed (*n*=3 independent experiments)

The above results confirm the ability of SAL and the other two potassium ionophores to trigger apoptosis in lymphocytes. The property of these ionophores to accumulate in the mitochondria suggested that they might induce apoptosis by altering organellar potassium homeostasis via direct action at the level of the IMM. If this hypothesis were correct, death induced by these agents ought to be independent of Bax and Bak. These two pro-apoptotic proteins become inserted into the outer mitochondrial membrane during apoptosis and contribute to cytochrome *c* release from the intermembrane space, which however occurs also via other pathways.^23, 24, 25^ Loss of cytochrome *c* has been shown to occur upon SAL treatment at later stages of apoptosis. We used WT and Bax/Bak double-knockout (DKO) mouse embryonic fibroblasts (MEFs) and observed that the absence of Bax and Bak did not confer protection against SAL, NIG and VAL (Figure 2a and Supplementary Figure S3). As expected, MEF DKO cells were instead more resistant to staurosporine.

Altogether these data suggested that apoptosis induced by these three ionophores might be, at least in part, due to their direct effect on mitochondria. Therefore, we measured acute changes of mitochondrial membrane potential (Δ*ψ*), production of ROS, matrix pH, swelling, mitochondrial morphology and respiration upon addiction of SAL, NIG and VAL. First, we observed that addition of 1 *μ*M SAL, similarly to NIG and in contrast to VAL, to isolated rat liver mitochondria (RLM) resulted in hyperpolarization (Figure 2b). Also in intact cells, only VAL caused depolarization (Figure 2c). Both depolarization and hyperpolarization might trigger ROS release (e.g. Zorov *et al.*^26^), and an increase in intracellular ROS 30 h after SAL addition to intact cells was observed.^13^ In B-CLL cells, on a time scale of minutes, only VAL induced a significant mitochondrial ROS release measured by MitoSOX; even at 10 *μ*M SAL, ROS production was comparable to that of untreated cells (Figure 2d). The same result was obtained in Jurkat lymphocytes (not shown), and in our study, preincubation with membrane-permeant ROS scavengers PEGylated catalase and PEGylated superoxide dismutase did not affect SAL-induced apoptosis, while it did reduce VAL-associated death (Supplementary Figure S4).

**Figure 3:**
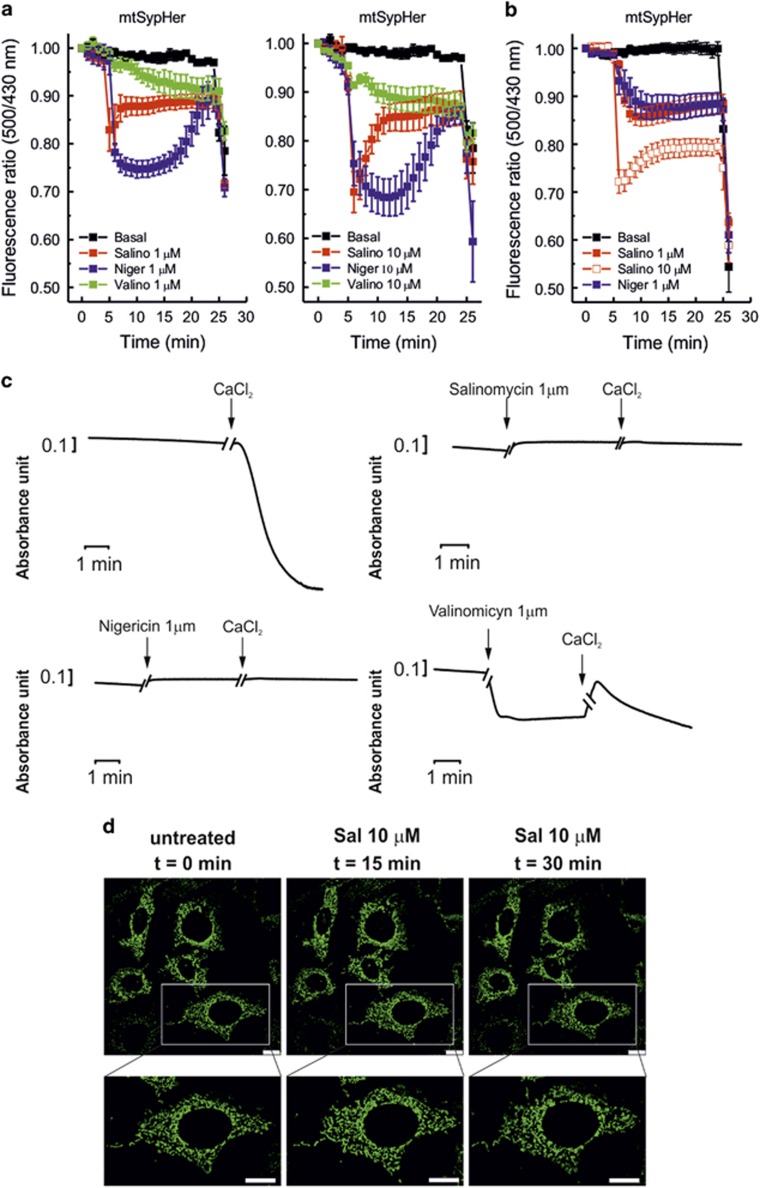
Salinomycin induces an instantaneous acidification of matrix pH in intact cells. (**a** and **b**) Measurement of transient variation of mitochondrial matrix pH in MEF WT cells (**a**) and in HMLE-Twist cells (**b**) expressing mito-SypHer; changes in pH correspond to variations in the 535-nm fluorescence emission after alternative excitation at 405 and 488 nm. Results are expressed as mean 500/430 nm ratios ± S.E.M. of four different experiments. Addition of drugs is indicated by red arrow, while Na-acetate (NaAc) was used as positive control (blue arrows). (**c**) Isolated rat liver mitochondria (RLM) swelling was measured as reduction of mitochondrial absorbance over time at 540 nm. CaCl_2_ (140 *μ*M) was used to induce PTP opening and swelling. (**d**) MEF WT cells expressing mito-YFP were treated with SAL at the indicated concentrations and live images were acquired over time to monitor changes in mitochondria morphology. Bars correspond to 25 *μ*m. Magnified images are shown in the lower row. Results shown in (**a**)–(**d**) are representative of three independent experiments

Given the similar consequences of NIG and SAL addition, we presumed that SAL mediates exchange of protons with potassium. To directly prove this point, matrix pH was measured in intact cells using mitochondria-targeted SypHer.^27^ At 1 and 10 *μ*M applied concentrations, both NIG and SAL triggered an immediate matrix acidification in MEF cells with a subsequent alkalinization that did not, however, re-establish the original pH value (Figure 3a). As mentioned above, SAL was shown to act on tumor stem cells as well, with a lower IC_50_ than the one found here for Jurkat, MEF or B-CLL cells.^1^ In particular, Gupta *et al.*^1^ showed (and we confirmed (not shown)) that immortalized human mammary epithelial cells (HMLE) ectopically expressing the transcription factor Twist and therefore forced to undergo epithelial–mesenchymal transition are sensitive to SAL, displaying an IC_50_ of around 1 *μ*M regarding cell viability. Therefore, we asked whether SAL shows the same effect on the mitochondria of cells with stem cell-like features and used HMLE-Twist cells to measure mitochondrial function. As Figure 3b illustrates, sustained matrix acidification upon SAL and NIG addition could be observed also in these cells. ROS release and changes in membrane potential (Supplementary Figure S5 and S6) occurred similarly to the other cells described above.

Matrix acidification is due to the entry of protons in exchange for potassium ions leaving the matrix. VAL has also been shown to cause acidification to some extent in the absence of K^+^/H^+^ exchanger inhibitors.^28^ Given that acidification is known to prevent activation of the permeability transition pore (PTP) (e.g. Szabo *et al.*^29^ and Bernardi *et al.*^30^), which causes swelling and might contribute to cytochrome *c* release,^24^ we tested whether SAL prevents calcium-induced PTP opening. A decrease of the absorbance, indicative of swelling, can be detected in isolated mitochondria upon addition of 140 *μ*M calcium, sufficient to trigger the PTP opening (Figure 3c). In contrast, SAL and NIG, as expected, completely abolish the swelling due to calcium-induced PTP opening, at least on a relatively short time scale, even though mitochondria are able to take up calcium even in the presence of SAL as assessed by calcium retention assay (not shown). Addition of VAL results in incomplete swelling presumably due to entry of potassium and to the subsequent compensating activation of the K^+^/H^+^ antiporter.^31^ Rapid changes in mitochondrial morphology upon addition of VAL were observed also in intact cells using mitochondria-targeted green fluorescence protein (Dimmer *et al.*^32^ and Supplementary Figure S7). These experiments indicated that although SAL and NIG affect to a small extent the mitochondrial network, they do not have drastic early effects similar to those induced by VAL (massive swelling; Figure 3d and Supplementary Figure S7).

**Figure 4:**
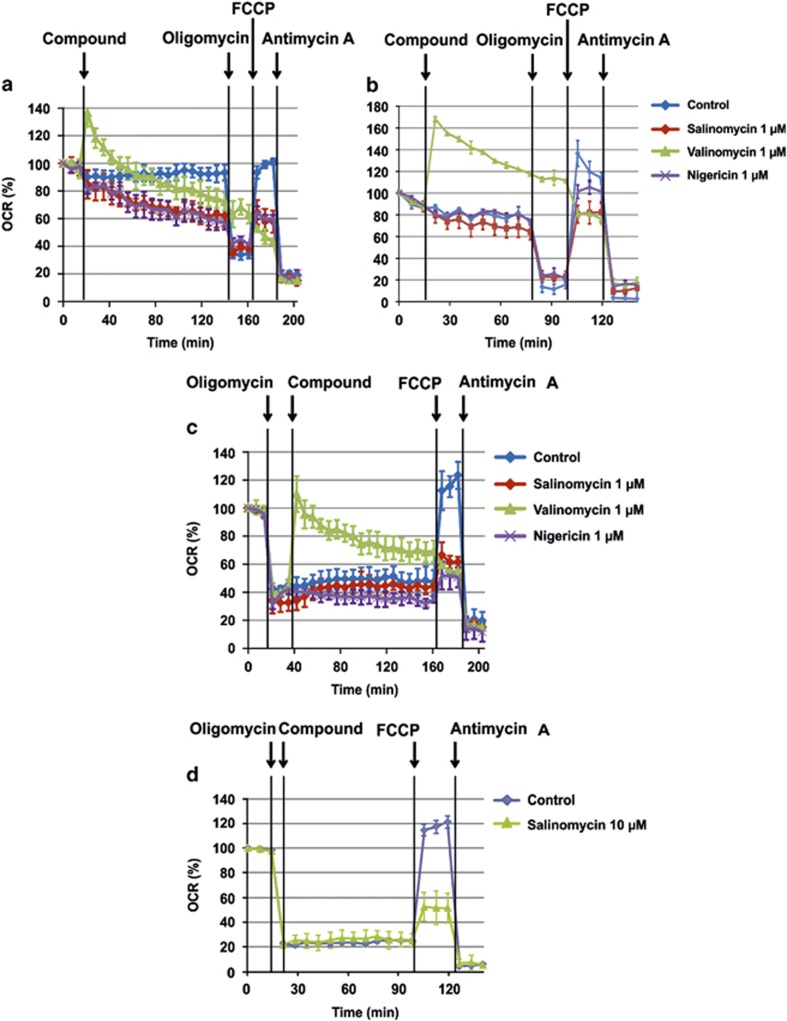
Salinomycin and nigericin affect respiration in a similar manner. (**a**–**d**) Oxygen consumption rate (OCR) of MEF WT cells (**a**, **c** and **d**) and of HMLE-Twist cells (**b**) was measured in the presence of 1 *μ*M salinomycin, nigericin and valinomycin (compounds). Representative experiments are shown. The compounds were added to the cells either before (**a** and **b**) or after (**c** and **d**) inhibition of ATP synthase activity with oligomycin. The effect of 10 *μ*M SAL in MEF cells was comparable to that of 1 *μ*M, indicating that already the lower concentration exerts the maximal effect

Although mitochondrial depolarization, ROS production and swelling do not take place in the early phases following SAL and NIG addition, the matrix acidification is expected to severely impact on mitochondrial bioenergetics. Adenosine triphosphate (ATP) depletion in cells upon SAL addition has been described after 24 h,^10^ but whether it occurs as a direct consequence of SAL action on mitochondrial respiration or as a secondary independent effect is unclear. Figure 4 illustrates that already 1 *μ*M SAL and NIG significantly reduced basal respiration in intact adherent MEFs as assessed by the extracellular flux analyzer using a previously established protocol.^33^ This decrease in respiration was further accentuated by oligomycin, which blocks the ATP synthase. Subsequent addition of the uncoupler carbonylcyanide-*p*-trifluoromethoxyphenylhydrazone (FCCP) did not restore respiration, whereas the addition of antimycin A, an inhibitor of complex III, completely abolished oxygen consumption in both MEFs (Figure 4a) and HMLE-Twist cells (Figure 4b). Antimycin A inhibits the oxidation of ubiquinol in the electron transport chain of oxidative phosphorylation (OxPhos), preventing thereby the formation of the proton gradient across the IMM. In summary, SAL and NIG drastically reduced the respiratory response to the addition of FCCP. In contrast, VAL induced an immediate increase in respiration and a loss of response to oligomycin. When cellular respiration in MEFs was decreased by oligomycin, the addition of VAL but not of SAL and NIG induced a recovery of the respiratory rate (Figure 4c). This recovery is a reflection of the loss of Δ*ψ*m induced by VAL. Subsequent addition of FCCP had no effect, whereas antimycin A reduced respiration (Figure 4c). Similar changes were observed in both settings with 10 *μ*M SAL (Figure 4d), indicating that at least in MEF cells, 10 *μ*M SAL was sufficient to drastically reduce bioenergetic efficiency. These results strongly suggest that matrix-pH-perturbing ionophores significantly reduce respiratory efficiency, contributing to the observed cell death.

**Figure 5:**
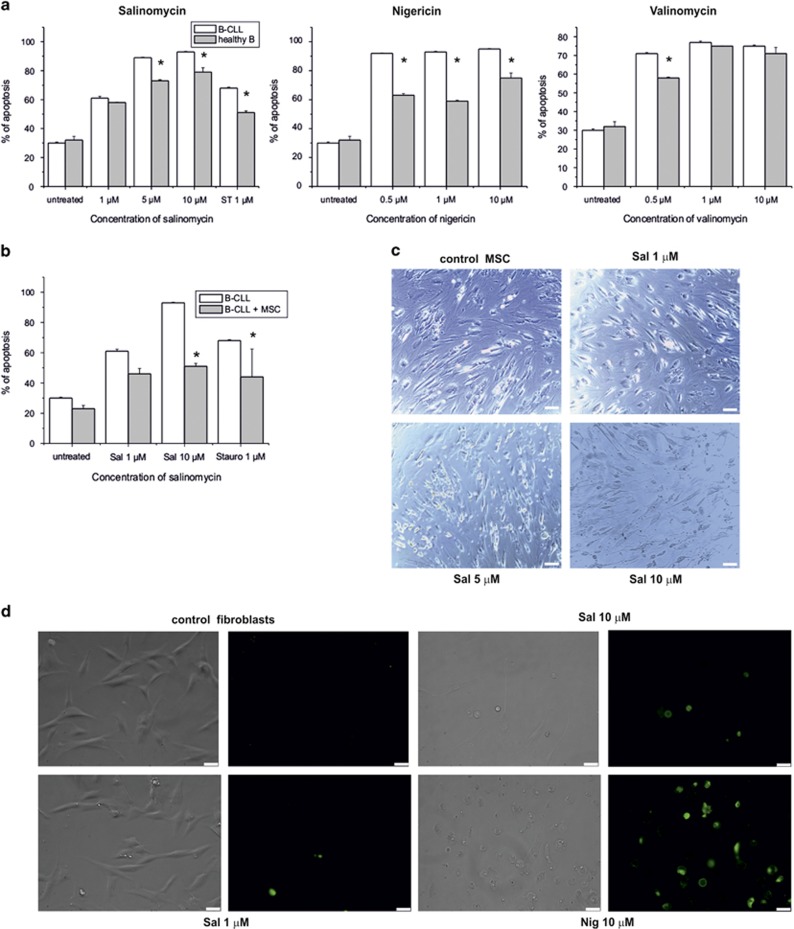
Salinomycin, nigericin and valinomycin affect survival of leukemic B cells, mesenchymal stromal cells and fibroblasts. (**a**) Comparison of apoptosis in pathologic B-CLL cells and B cells from healthy subjects (cells were incubated for 24 h in the presence of the indicated compounds and cell death was determined by FACS by staining with FITC-Annexin V and propidium iodide). Quantification of the cell death (all annexin-positive cells) ± S.E.M. (*n*=14 for B-CLL cells and *n*=6 for healthy B cells). (**b**) Apoptosis of B-CLL (*n*=14) cells compared with death occurring in the pathologic cells co-cultured with MSC (*n*=4). Apoptotic cells were identified by FACS as in (**a**). (**c**) Mesenchymal stromal cells were photographed after 24 h treatment with SAL and removal of co-cultured B-CLL cells by washing. Bars correspond to 75 *μ*m. At higher concentrations of SAL the number of cells decreased and the cells showed significant morphological alterations. (**d**) Human primary fibroblasts treated with the indicated substances for 24 h. Annexin-FITC binding is shown. Results shown in (**c**) and (**d**) are representative of three independent experiments. Bars correspond to 50 *μ*m

In light of the above results we then compared B-CLL cells directly with B lymphocytes isolated from healthy subjects as previously described.^19^ Under the experimental conditions used, SAL-induced apoptosis not only in B-CLL cells but also in healthy B cells, although to a statistically significantly less extent than in pathologic cells (Figure 5a). The same experiments were performed on B-CLL cells co-cultured with mesenchymal stromal cells (MSCs) from healthy donors,^34^ since MSCs of the bone marrow microenvironment have been reported to protect B-CLL cells from apoptosis induced by conventional chemotherapeutics.^35^ The apoptosis-inducing effect of SAL was significantly reduced by the presence of MSCs both for pathologic (Figure 5b) and for healthy B cells (not shown) in agreement with the notion that adhesion of B-CLLs on MSC favors survival^36^ and MSCs protect leukemia cells from apoptosis induced by chemotherapeutic drugs.^37^ SAL was toxic to MSC after 24 h of treatment both when co-culturing B-CLL with MSC (Figure 5c and Supplementary Figure S8) and when culturing MSC alone (Supplementary Figure S9). In order to test also another non-tumoral system, cultured primary human skin fibroblasts were used (Figure 5d). Again, these cells were sensitive to SAL and were even more apoptotic with NIG applied at 10 *μ*M concentration.

## Discussion

In the present work, by comparing the effects of three K^+^ ionophores on isolated mitochondria as well as in intact cells, we demonstrate that SAL and NIG, two drugs identified as promising chemotherapeutics, both act as K^+^/H^+^ exchangers at the level of the IMM and contribute to the loss of mitochondrial function in intact cells.

Our data clearly show that SAL action is comparable to that of NIG while being distinct from the effects of VAL, and demonstrates that in intact cells SAL mediates K^+^/H^+^ exchanger activity also at the level of the IMM. The measurements in cells indicating IMM hyperpolarization, mitochondrial matrix acidification and decrease of respiration leave no doubt that, similarly to NIG, SAL reaches the mitochondria within a few seconds upon addition. Indeed, NIG has been reported also by other groups to cause IMM hyperpolarization, without causing an acute effect on the cytosolic pH or on the plasma membrane potential,^38, 39^ and SAL was shown to cause extensive damage to the mitochondria of *Eimeria* sporozoites.^40^ Our results suggest that the reported ATP depletion upon SAL addition is a result of the decreased respiration and that increase in mitochondrial ROS release and mitochondrial depolarization detected 24 h after addition are secondary effects occurring at a later time point. These ionophores impact also cellular ion homeostasis, that is, SAL suppresses autophagy flux and lysosomal proteolytic activity.^41^ NIG and SAL may mediate the export of potassium through the plasma membrane down its concentration gradient in exchange for the uptake of protons, and as a consequence cause acidification of the cytosol. However, it has been shown that 0.5 *μ*M NIG did not alter the cytosolic pH under conditions that caused a pronounced acidification of the mitochondrial matrix within 15 min.^38^ On the other hand, on a longer time scale SAL treatment has been shown to cause membrane scrambling.^42^

In mitochondria, NIG and SAL cause hyperpolarization, that is, a shift of Δ*ψ* from the resting potential toward more negative values, since by acting as H^+^/K^+^ exchangers, they adjust ΔpH according to the K^+^ gradient across the IMM (depletion of K^+^ during organelle isolation does not occur (e.g. Szabo and Zoratti^25^ and Bernardi^31^): the equilibrium condition for K^+^/H^+^ electroneutral exchange is Δ*μ*_K_=–Δ*μ*_H_. Since K^+^ is by far more concentrated than H^+^, upon addition of NIG or SAL the Δ*μ*_H_ will essentially tend to the value set by the Δ*μ*_K_ (with the opposite orientation). Δ*ψ* increases to maintain a constant Δ*μ∼*_H_ in spite of the decrease (or inversion) of Δ*μ*_H_.^38, 43^ In other words, proton influx being in exchange for K^+^, that is, electroneutral, does not have by itself any effect on potential. Hyperpolarization derives from the collapse of ΔpH, which the mitochondrion compensates by increasing Δ*ψ* so as to keep the overall electrochemical potential nearly constant. Neither NIG nor SAL increases mitochondrial ROS production in intact cells, since these agents do not modify Δ*μ∼*_H_ on short-term scale; instead, they cause matrix acidification. These results are in agreement with the study reporting that matrix alkalinization favors ROS generation, whereas matrix acidification induced by NIG strongly inhibits this process.^44^ The data presented here on VAL are also in line with the findings described in the literature.^31^

Mitochondrial matrix pH is a factor that controls OxPhos in intact cells. Matrix pH ranges from 7.7 to 8.2 in different cell types, while the pH of the cytosol is around 7.0. This proton gradient significantly contributes to the driving force on the ATP synthase and impacts several transport processes responsible for exchanging metabolites (e.g. pyruvate, glutamate) or ions between mitochondria and the cytosol (e.g. Greenbaum and Wilson^45^). Using a mitochondrial pH-sensitive probe (mtAlpHi), Akhmedov *et al.*^38^ observed in insulin-secreting cells a matrix acidification using 0.5 *μ*M NIG, which was partially recovered over the following 15 min, similarly to our observations in MEF cells. Matrix realkalinization depends on the operation of the H^+^ pumps and on the electrogenic influx of cations and efflux of anions,^46^ but a ΔpH of 0.4 unit persisted after the partial recovery in Akhmedov *et al.*^38^. In addition, matrix acidification slowed down mitochondrial ATP synthesis by 20% over 20 min despite hyperpolarization of the IMM, by regulating OxPhos downstream of Complex II. In the present study, a sustained decrease of respiration by addition of NIG and SAL is observed. This process might be compensated for a while by ATP hydrolysis due to the ability of the ATP synthase to work in the reverse mode, which presumably contributes to the ATP depletion observed (after 24 h) in several studies. Sustained decrease of the respiration is expected to lead also to loss of Δ*Ψ*m in the long term, which, in turn, might trigger autophagy, mitophagy and ROS release. Indeed, depolarization induced by FCCP leads to ROS release (e.g. Sassi *et al.*^47^) and is also known to trigger mitophagy (e.g. Lemasters^48^). Thus, the initial changes observed here upon SAL addition might well account for the published observations (mitochondrial depolarization, ATP depletion, ROS release and mitophagy^39^) occurring after >24 h. Caspase activation and cytochrome *c* release also occur at late time points.^9^ Whether cytochrome *c* can be released upon depolarization-induced PTP opening or due to accumulation of dysfunctional mitochondria remains to be determined.

The relationship between mitochondrial respiratory inhibition, ATP depletion and cell death may be dependent on the cell type as well as on specific conditions (e.g. Poltl *et al.*^49^). Importantly, sensitivity of the cells to agents affecting mitochondrial respiration depends on the metabolic state of the cells, those relying mostly on mitochondrial respiration rather than on glycolysis are more sensitive. Growing evidence indicates that several types of cancer cells (e.g. CSC-like surviving pancreatic ductal adenocarcinoma cells (PDAC)^50^ and both normal and leukemic stem cells^51^) are less glycolytic and more dependent on mitochondrial respiration than others. PDAC cells are unable to increase compensatory glycolytic fluxes following inhibition of OxPhos, thereby showing large sensitivity to inhibition of mitochondrial function. This may well explain the previously reported selectivity of SAL towards CSCs. Thus, differences in the sensitivity of the cell types used here and in other works towards SAL can likely be ascribed to differences in metabolic state. Healthy human fibroblasts and B lymphocytes have been shown to principally rely on OxPhos for their metabolism,^52^ possibly accounting for the SAL effect observed here. In accordance with our findings, SAL was found to be cytotoxic to healthy human peripheral blood lymphocytes at 10 *μ*M concentration.^53^ In general, induction of mitochondrial dysfunction and related energy metabolism^54, 55, 56^ and/or of substantial ROS release^57, 58^ is considered a valid strategy to fight malignant diseases. The question also arises whether differences in sensitivity to SAL between CSCs and non-CSCs might be ascribed to differences in the mitochondrial response to SAL. Our data suggest that this is not the case, since the observed effects equally take place in the two cell types. Instead, it has been reported that autophagy flux is essential for maintaining proliferation of CSCs and that SAL reduces the activity of cathepsins resulting in the inhibition of lysosomal activity and autophagic flux to a greater extent in CSCs with respect to non-CSC cells.^41^

In summary, in the present work we provide evidence that SAL functions in intact cells as an K^+^/H^+^ antiporter and directly impacts mitochondrial function in a few minutes upon addition. Our findings contribute to the understanding of the mechanism of action of this promising anticancer drug. The results strengthen the hypothesis that polyether ionophore antibiotics contribute to apoptosis induction by a common mechanism and might explain toxicity upon SAL treatment (e.g. Boehmerle *et al.,*^5^ Scherzad *et al.,*^53^ and Ojo *et al.*^59^). Further studies will be necessary to exactly delineate the factors contributing to the moderate to high selectivity of SAL and NIG toward tumoral cells as observed in several cases, given that these ionophores *a priori* impact mitochondrial functions independently of differences in signaling pathways (e.g. Wnt) and in redox state and of the mitochondrial hyperpolarization typical of tumoral cells. Therefore, the hypothesis that the apoptotic effects of SAL and NIG depend on the ability of the cells to compensate mitochondrial dysfunction, deserves to be tested in future studies. For example, peroxisome proliferator-activated receptor gamma coactivator 1-*α*, an important regulator of mitochondrial biogenesis that is also involved in the regulation of several stress programs and might integrate mitochondrial biogenesis into cellular stress adaption, might be involved in compensatory mechanisms (e.g. Hofer *et al.*^60^).

## Materials and Methods

### Reagents and cell lines

SAL, NIG and VAL and other commercial chemicals were purchased from Sigma-Aldrich (Milan, Italy) unless otherwise specified.

Mouse embryonic fibroblast (MEF), MEF WT, BAX/BAK-less (DKO) and mito-YFP cells were grown in Dulbecco's modified Eagle's medium (DMEM) additionated with 10 mM HEPES buffer (pH 7.4), 10% (v/v) fetal bovine serum (FBS), 100 U/ml penicillin G, 0.1 mg/ml streptomycin and 1% non-essential amino acids (100 × solution; Life Technologies, Thermo Fisher Scientific Inc, Waltham, MA, USA), in a humidified atmosphere of 5% CO_2_ at 37 °C. Jurkat T lymphocytes were grown in RPMI-1640 supplemented as above. B cells were isolated and maintained in culture on a MSC layer as previously reported.^34^ Briefly, 2 × 10^5^/well CLL MSCs were seeded and incubated for a few days before the experiment at 37 °C in 5% CO_2_ up to confluence. Then purified B-CLL cells (CD19^+^/CD5^+^) were added to the MSC layer at a ratio of 2–5 : 1. Cells were then treated with SAL, NIG or VAL for 24 h to evaluate a possible resistance to this agent due to the co-culture. Human skin fibroblasts, kindly provided by Prof. V Bianchi (Padova), were cultured in DMEM supplemented with 10% FBS, l-glutamine and streptomycin.

HMLE TWIST cells were a kind gift of Prof. Weinberg and were grown in DMEM F1/2 supplemented with insulin, hydrocortisone, bovine pituitary extract and epidermal growth factor (all from Life Technologies, Thermo Fisher Scientific Inc). Cells were grown and cultured as reported in Cordenonsi *et al.*^61^

### Proliferation and cell death assays

To determine cell viability, tetrazolium reduction (MTT) assay was used as reported previously.^18^ Briefly, 0.005 × 10^6^ MEF WT or DKO cells were seeded in standard 96-well plates in 200 *μ*l of DMEM for 24 h. Then, the medium was replaced with DMEM without phenol red (PR) and FBS and cells were treated as indicated in the figure legends. Suspension cells were seeded and treated in DMEM without PR and FBS. After incubation, CellTiter 96 AQUEOUS One solution (Promega, Milan, Italy) was added to each well as indicated by the supplier. Absorbance at 490 nm to detect formazan formation was measured using a Packard Spectra Count 96-well plate reader (Life Technologies, Thermo Fisher Scientific Inc).

Apoptosis for B-CLL and healthy cells was determined by FACS analysis (FACS Canto II, BD BioSciences, Franklin Lakes, NJ, USA) using a double staining with Annexin V-FLUOS (Roche, Basel, Switzlerland) and propidium iodide (PI; Sigma Aldrich, St. Louis, MO, USA) as reported before.^19^ After incubation with the different drugs, cells were incubated with PI (final concentration 1 *μ*g/ml) and Annexin-V-FLUOS (0.6 *μ*l/sample) for 20′ at 37 °C in the dark, and were analyzed by FACS. For human skin fibroblasts, cells were seeded in a 24-well plate and treated for 24 h as indicated in 1 ml of DMEM without PR and FBS. After incubation, 2 *μ*l/well of Annexin V-FITC was added and cells were incubated for 20′ at 37 °C in the dark, and were analyzed by a Leica DMI 4000 fluorescence microscope (Leica Microsystems, Wetzlar, Germany).

### Mitochondrial morphology

Mitochondrial network was studied by using MEF mito-YFP cells seeded directly onto glass coverslips in six-well plates (0.05 × 10^6^) in DMEM. Then, coverslips were mounted onto holders, washed, filled with 1 ml of DMEM without PR and FBS and compounds were added directly onto the holder on the microscope stage of a Leica SP5 confocal system mounted on a Leica DMI6000 microscope.

### Determination of mitochondrial matrix pH in live cells

MEF WT cells were seeded at a density of 50 × 10^3^ cells/well onto glass coverslips. pH measurements were performed in Ca^2+^/Mg^2+^ supplemented HEPES buffer (HBSS, Invitrogen, Milan, Italy) 24 h after transfection with a mtSypHer expression vector using Lipofectamine 2000 (Invitrogen). Coverslips were mounted onto the holder and the different compounds were added directly and analyzed by a Leica SP5 confocal system mounted on a Leica DMI6000 microscope. Ratiometric sequential images of the 535 nm emission fluorescence were acquired every 3 min during 30 min with a × 63 objective and the LAS-AF software after alternative excitation at 430 nm and 500 nm. As a control of acidification, cells were treated with 30 mM NaAc at the end of each experiment. The multi-measure plug-in of Image J software (NIH) was used to estimate mean fluorescence ratios of selected ROIs matching the mitochondria in at least four experiments following background subtraction; results are expressed as mtSypHer (500/430 nm) ratio.

### Mitochondrial membrane potential and ROS production

Mitochondrial membrane potential (MMP) and ROS production were measured as in Leanza *et al.*^19^ Briefly, MMP was monitored using tetramethylrhodamine methyl ester (TMRM; 20 nM), while ROS production was assayed using MitoSOX (1 *μ*M). B cells either from CLL patients or from healthy subjects were incubated for 20 min at 37 °C. After incubation, the indicated compounds were added and the decrease in TMRM fluorescence or the increase in MitoSOX fluorescence was measured by FACS. HMLE TWIST cells (0.07 × 10^6^ cells/well) were seeded in a 12-well plate in 1 ml of their culture medium. The day after, cells were incubated with TMRM 20 nM and MitoSOX 1*μ*M in HBSS (Life Technologies, Thermo Fisher Scientific Inc) for 20 min at 37 °C in the dark. After incubation, compounds were added as indicated in the figure and the decrease in TMRM fluorescence or the increase in MitoSOX fluorescence was measured by a Leica DMI 4000 fluorescence microscope at the indicated time points.

### Oxygen consumption assay

Oxygen consumption by adherent cells was measured using an XF24 Extracellular Flux Analyzer (Seahorse Bioscience, North Billerica, MA, USA) as reported before.^33, 47^ MEF WT cells were seeded at 3 × 10^4^ cells/well in 200 *μ*L of supplemented culture medium (DMEM; Sigma Aldrich). Oxygen consumption rate (OCR) was measured at preset time intervals while the instrument automatically carried out the preprogrammed additions of the various compounds (oligomycin final concentration 1 *μ*g/ml, FCCP 400 nM, antimycin A 1 *μ*M), added as a solution in 70 *μ*L of DMEM. HMLE TWIST cells were seeded at 6 × 10^4^ cells/well in 200 *μ*L of supplemented culture medium. As for MEF WT, OCR was measured at preset time intervals upon the addition of the various compounds (oligomycin final concentration 0.5 *μ*g/ml, FCCP 1 *μ*M, antimycin A 1 *μ*M), added as a solution in 70 *μ*L of DMEM. A massive loss of cells because of death and detachment was excluded by direct microscopic observation of the cells at the end of the experiments (not shown).

### RLM isolation and determination of RLM membrane potential and swelling

Rats were killed and the liver was removed and immediately immerged in an ice-cold isolation medium (250 mM sucrose, 5 mM Hepes, 2 mM EGTA, pH 7.5). The liver tissue was minced, thoroughly rinsed several times with ice-cold medium and then homogenized in the same buffered solution using a glass/Teflon Potter homogenizer. Mitochondria were then isolated by conventional differential centrifugation and the protein content was measured by the biuret method with bovine serum albumin as a standard.

Experiments were carried out with 1 mg of mitochondrial protein/ml suspended in a standard incubation medium containing 200 mM sucrose, 10 mM HEPES (pH 7.4), 5 mM Na-succinate, 1.25 *μ*M rotenone and 1 mM phosphate, additionated with 2 mM KCl.

Membrane potential (Δ*ψ*) was determined on the basis of the distribution of lipid-soluble cation tetraphenylphosphonium (TPP^+^) across the mitochondrial membrane, measured by a TPP^+^-specific electrode, as in Kamo *et al.*^62^

Mitochondrial swelling was determined by measuring light scattering at 90° with a Perkin Elmer LS50B spectrofluorometer at 540 nm with a 5.5-nm slit width.

## Abbreviations

ATP: adenosine triphosphate
B-CLL: B-cell chronic lymphocytic leukemia
CSC: cancer stem cell
FCCP: carbonylcyanide-*p*-trifluoromethoxyphenylhydrazone
HMLE: human mammary epithelial cell line
IMM: mitochondrial inner membrane
MEF: mouse embryonic fibroblast
MSC: mesenchymal stromal cells
MTT: (3-(4,5-dimethylthiazol-2-yl)-2,5-diphenyltetrazolium bromide)
NIG: nigericin
OxPhos: oxidative phosphorylation
PDAC: pancreatic ductal adenocarcinoma
PTP: permeability transition pore
ROS: reactive oxygen species
SAL: salinomycin
VAL: valinomycin
