# Supplementary material for: Early effects of the antineoplastic agent salinomycin on mitochondrial function
Source: Cell Death Dis. 2015 Oct 22;6(10):e1930–. doi: 10.1038/cddis.2015.263 (PMC4632293; doi:10.1038/cddis.2015.263)
Supplement: Supplementary Information [file cddis2015263x1.pdf]

Figure S1

Salinomycin

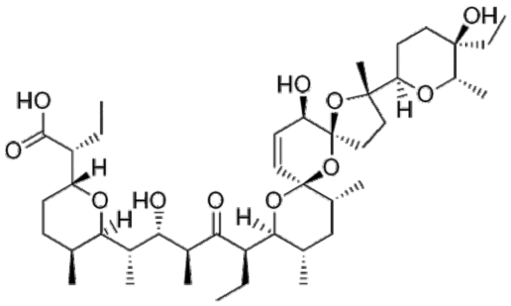

Valinomycin

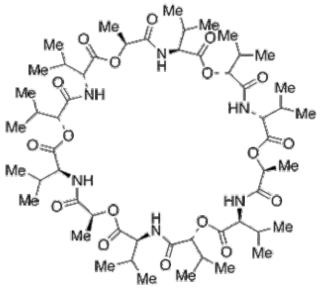

Nigericin

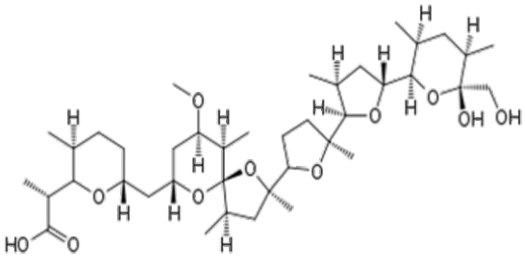

Figure S2

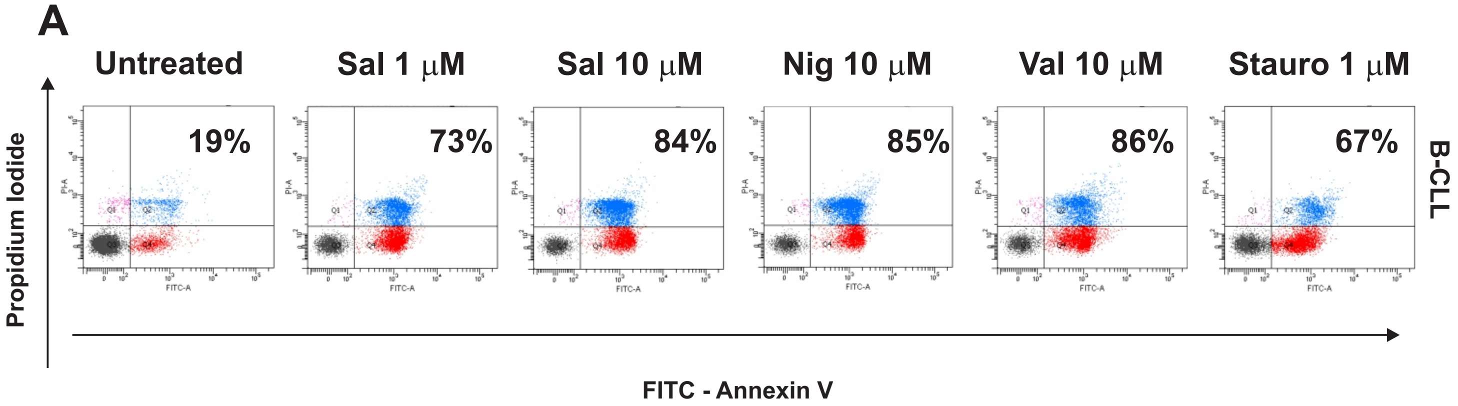
A
Untreated
Sal 1 μM
Sal 10 μM
Nig 10 μM
Val 10 μM
Stauro 1 μM
19%
73%
84%
85%
86%
67%
B-CLL
Propidium Iodide
FITC - Annexin V

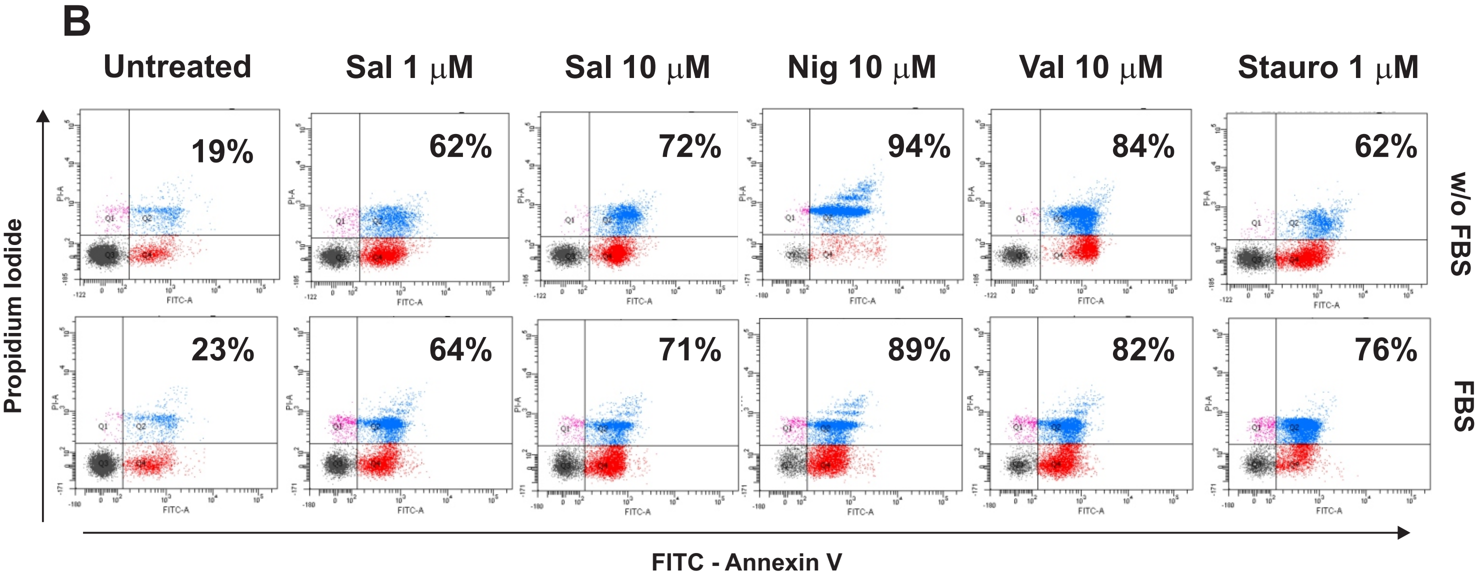
B
Untreated
Sal 1 μM
Sal 10 μM
Nig 10 μM
Val 10 μM
Stauro 1 μM
19%
62%
72%
94%
84%
62%
w/o FBS
23%
64%
71%
89%
82%
76%
FBS
Propidium Iodide
FITC - Annexin V

Figure S3

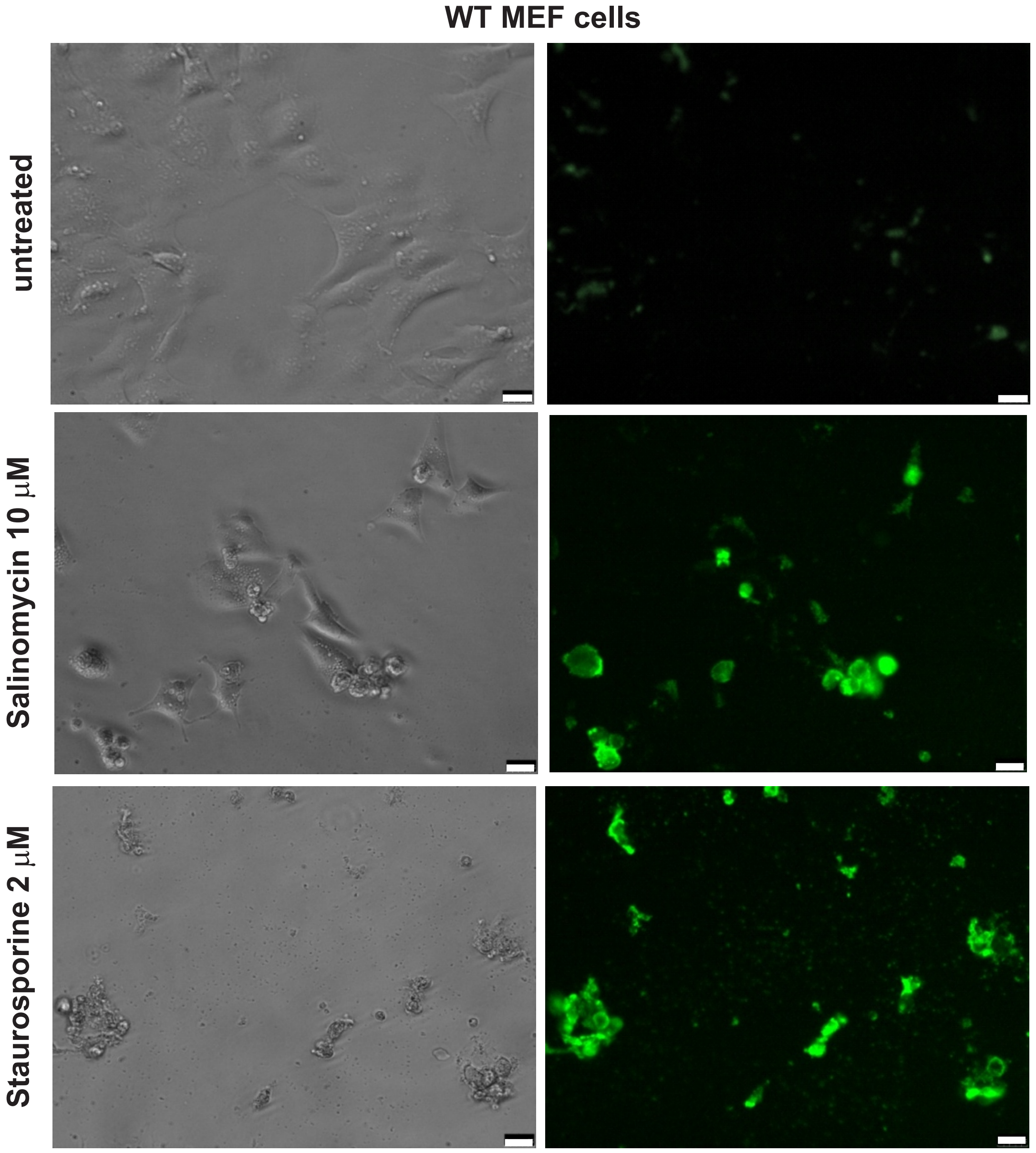

WT MEF cells
untreated
Salinomycin 10 μM
Staurosporine 2 μM

Figure S4

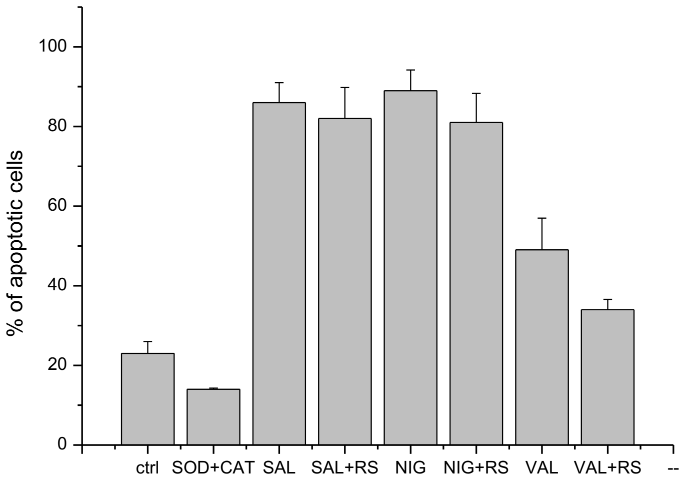

% of apoptotic cells
100
80
60
40
20
0
ctrl
SOD+CAT
SAL
SAL+RS
NIG
NIG+RS
VAL
VAL+RS
--

Figure S5

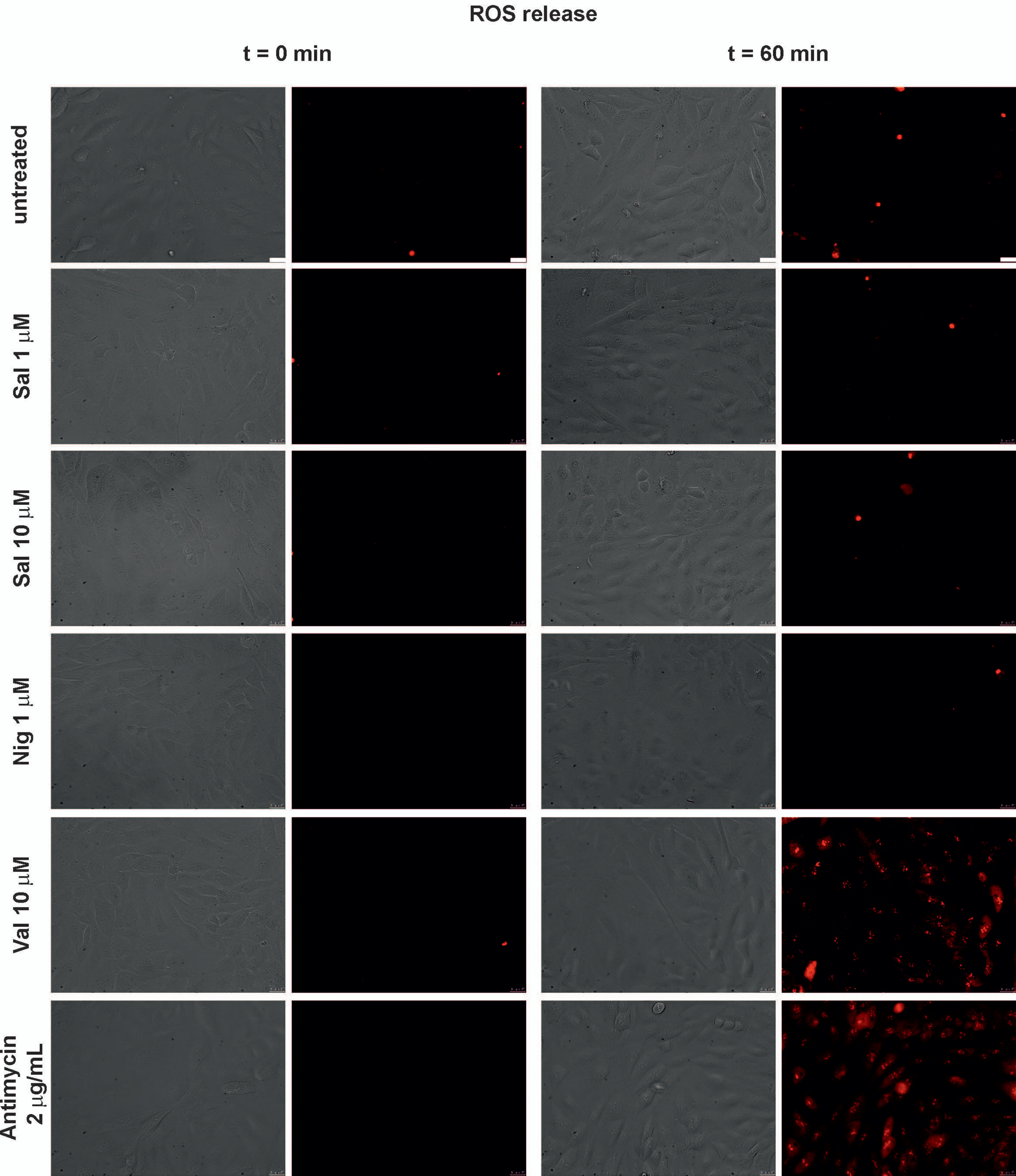
ROS release
t = 0 min
t = 60 min
untreated
Sal 1 μM
Sal 10 μM
Nig 1 μM
Val 10 μM
Antimycin
2 μg/mL

Figure S6

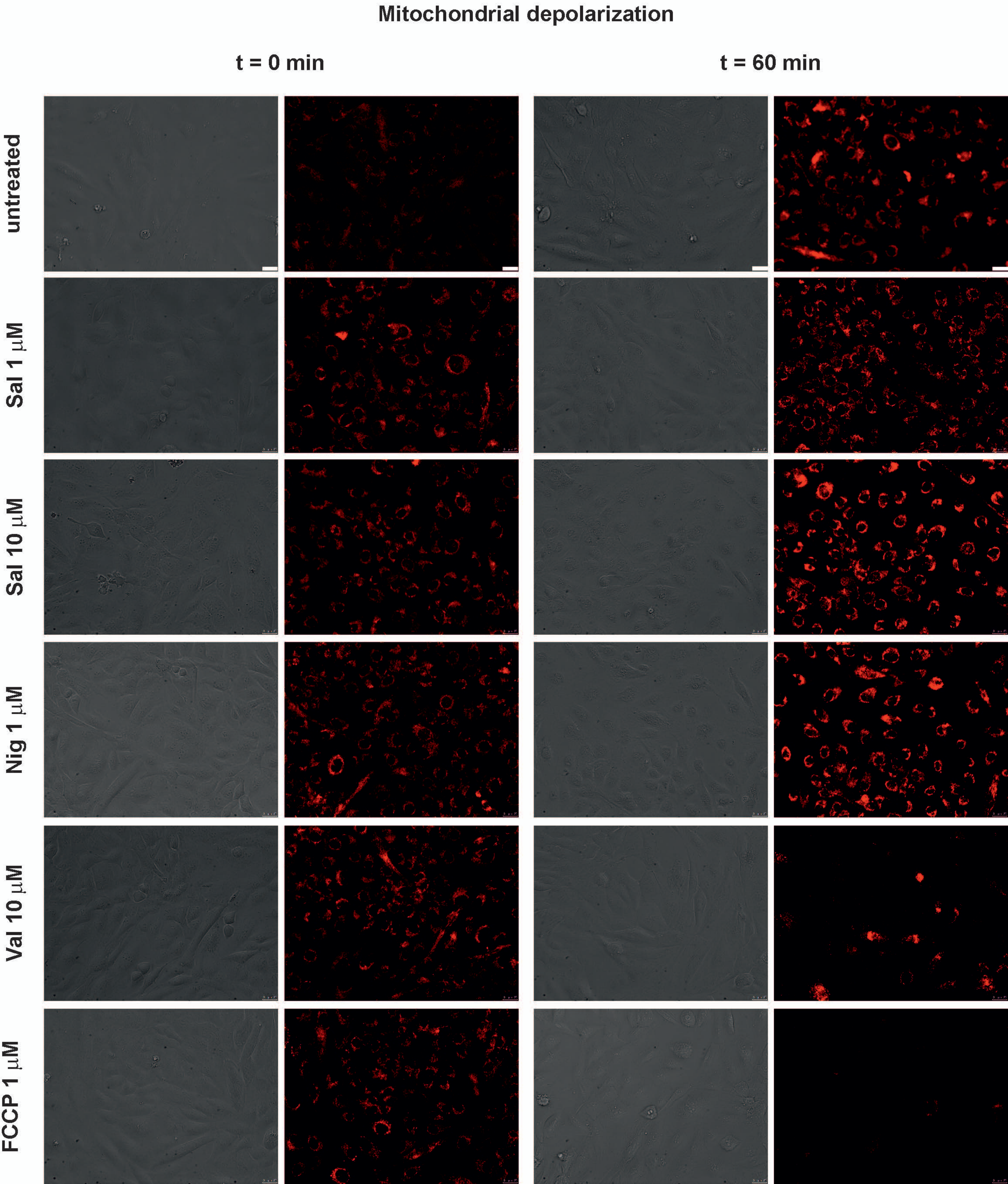
Mitochondrial depolarization
t = 0 min
t = 60 min
untreated
Sal 1 μM
Sal 10 μM
Nig 1 μM
Val 10 μM
FCCP 1 μM

Figure S7

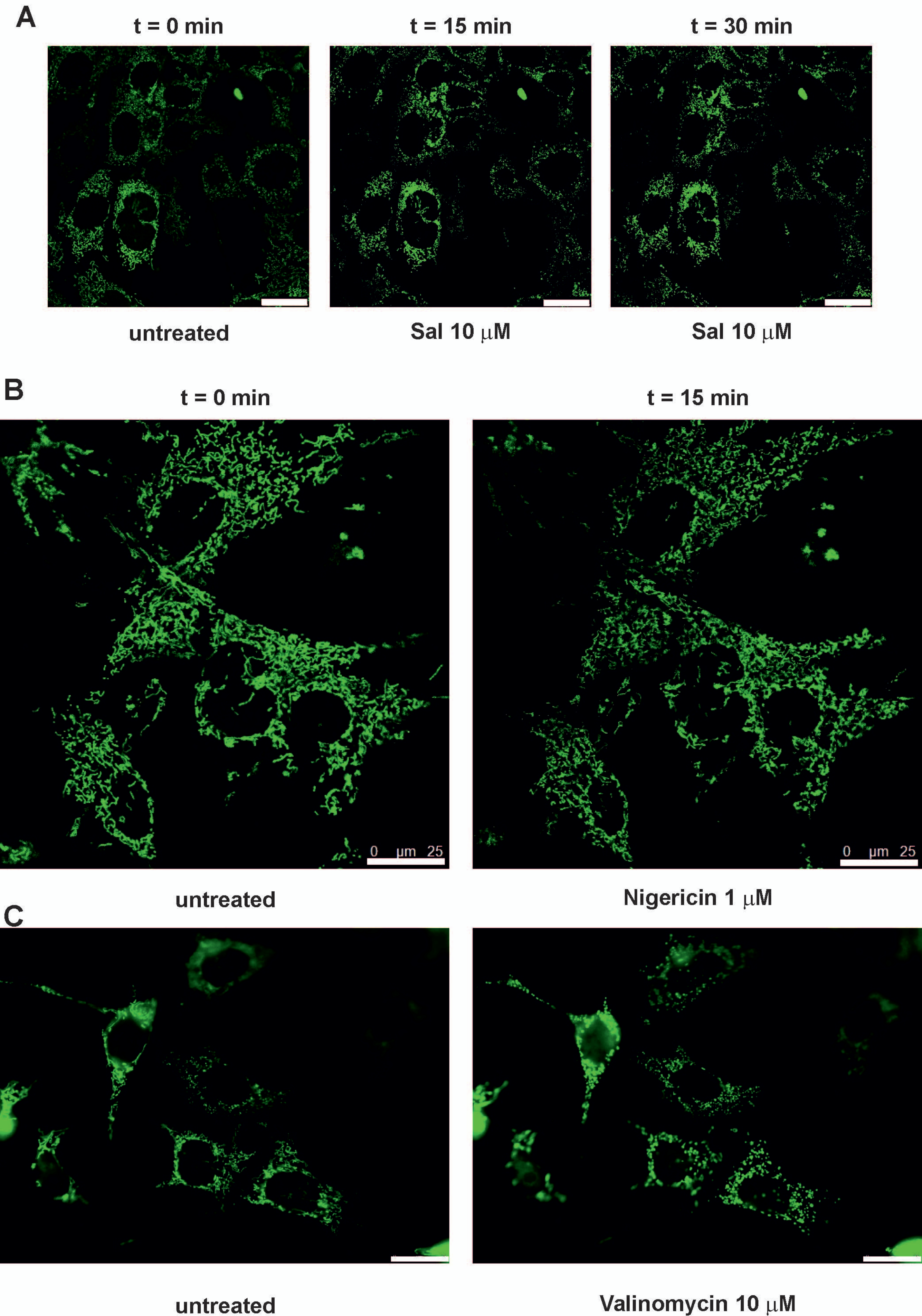
A
t = 0 min
t = 15 min
t = 30 min
untreated
Sal 10 μM
Sal 10 μM
B
t = 0 min
t = 15 min
0 μm 25
0 μm 25
untreated
Nigericin 1 μM
C
untreated
Valinomycin 10 μM

Figure S8

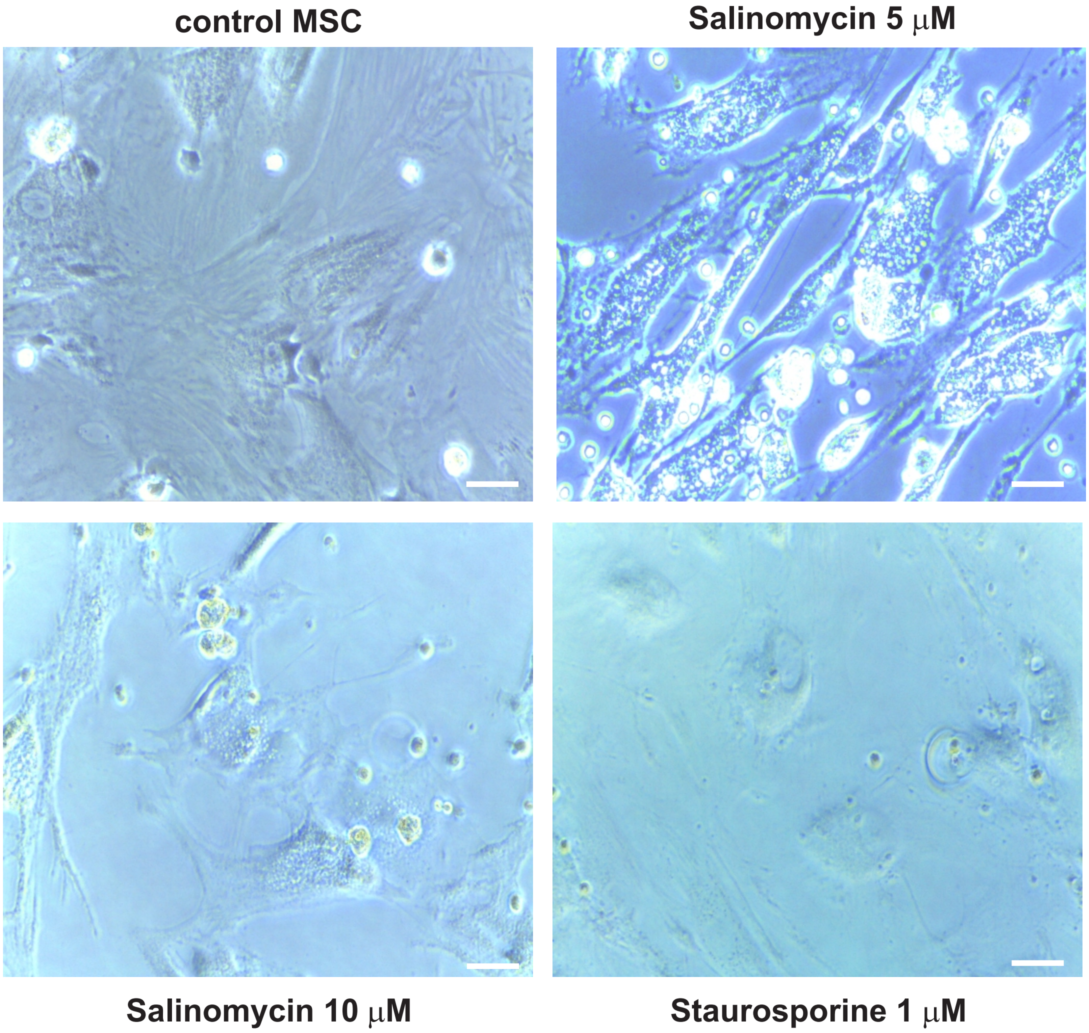
control MSC
Salinomycin 5 μM
Salinomycin 10 μM
Staurosporine 1 μM

Figure S9

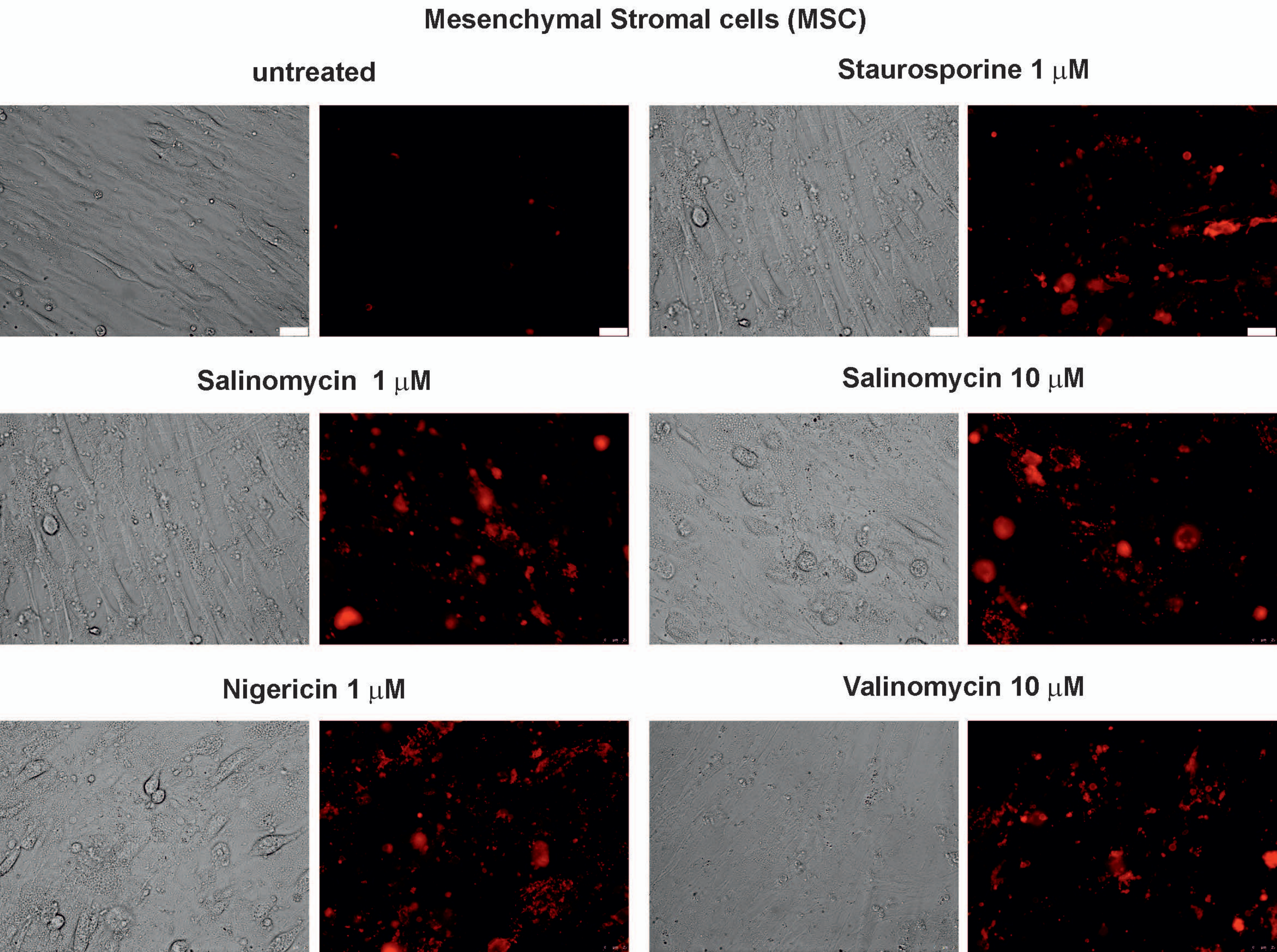
Mesenchymal Stromal cells (MSC)
untreated
Staurosporine 1 μM
Salinomycin 1 μM
Salinomycin 10 μM
Nigericin 1 μM
Valinomycin 10 μM
